# Changes in microcirculatory perfusion during cardiac surgery are paralleled by alterations in glycocalyx integrity

**DOI:** 10.1186/cc12150

**Published:** 2013-03-19

**Authors:** C Boer, NJ Koning, J Van Teeffelen, AB Vonk, H Vink

**Affiliations:** 1 VU University Medical Center, Amsterdam, the Netherlands; 2 Maastricht University, Maastricht, the Netherlands

## Introduction

Previous studies demonstrate that loss of glycocalyx integrity is associated with impaired microvascular function. We investigated whether glycocalyx dimensions are reduced in patients undergoing cardiac surgery with or without cardiopulmonary bypass (CPB), and are paralleled by loss of microcirculatory perfusion using *in vivo *microcirculation measurements.

## Methods

Patients undergoing on-pump surgery with nonpulsatile (*n = *11) or pulsatile (*n = *13) CPB or off-pump surgery (*n = *13) underwent sublingual sidestream dark-field imaging at baseline, during coronary grafting and upon ICU admission to assess perfused microvascular vessel density. Glycocalyx integrity was evaluated using the GlycoCheck Measurement Software, and expressed as the perfused boundary region (PBR). An increase in PBR represents deeper penetration of erythrocytes into the glycocalyx, and is indicative for compromised glycocalyx thickness.

## Results

The perfused vessel density remained stable during offpump surgery, while the PBR decreased from 2.6 ± 0.1 μm (baseline) to 2.3 ± 0.1 μm (ICU). Nonpulsatile CPB was associated with loss of microcirculatory perfusion during bypass (15.1 ± 2.7 mm/mm^2^) and ICU admission (15.3 ± 2.6 mm/mm^2^) compared with baseline (19.8 ± 2.8 mm/mm^2^). Pulsatile CPB reduced the perfused vessel density from 20.9 ± 2.4 to 16.7 ± 2.6 mm/mm^2^, but this was restored towards baseline levels upon ICU admission (20.3 ± 2.3 mm/mm^2^; *P *= 0.02 between groups). The PBR increased upon initiation of CPB in both groups, but remained elevated in the nonpulsatile flow group only. In the pulsatile group, the PBR started at 2.4 ± 0.1 μm, increased to 2.7 ± 0.1 μm during bypass but restored already during CPB towards a PBR value of 2.3 ± 0.1 μm (ICU). The PBR and perfused vessel density showed a good correlation (*r *= -0.65; *P *= 0.002), demonstrating loss of perfused vascular density when the glycocalyx is damaged.

## Conclusion

The glycocalyx is damaged after initiation of CPB, and restoration is impaired after exposure to nonpulsatile flow during the use of the heart-lung machine. A reduction in glycocalyx thickness, represented by an increased PBR, correlates with impairment of perfused microvascular vessel density. Our data support the use of intraoperative PBR monitoring as a novel clinical indicator of microcirculatory perfusion.

